# The COLIEE 2025 Competition on Legal Information Extraction and Entailment: Overview, Discussion, and Dataset Expansion

**DOI:** 10.1007/s12626-026-00199-9

**Published:** 2026-02-20

**Authors:** Randy Goebel, Yoshinobu Kano, Mi-Young Kim, Calum Kwan, Juliano Rabelo, Ken Satoh, Hiroaki Yamada, Masaharu Yoshioka

**Affiliations:** 1 Department of Computing Science and Alberta Machine Intelligence Institute (Amii), University of Alberta, Edmonton, AB Canada; 2 Faculty of Informatics, Shizuoka University, Hamamatsu, Shizuoka Japan; 3 Department of Science, Augustana Faculty, University of Alberta, Camrose, AB Canada; 4 Jurisage, Toronto, Canada; 5 Center for Juris-Informatics, ROIS-DS, Chiyoda-ku, Tokyo Japan; 6 Institute of Science Tokyo, Meguro-ku, Tokyo Japan; 7 Faculty of Information Science and Technology, Hokkaido University, Sapporo-shi, Hokkaido Japan

**Keywords:** COLIEE2025, Legal information retrieval, Legal information entailment

## Abstract

We summarize the 12th Competition on Legal Information Extraction and Entailment. In this edition, the competition included two tasks on case law, two tasks on statute law, plus a new pilot task on Tort law. The two case law components include an information retrieval task (Task 1), and the confirmation of an entailment relation between an existing case and an unseen case (Task 2). The statute law components include an information retrieval task (Task 3), and an entailment/question-answering task based on retrieved civil code statutes (Task 4). The new pilot task involves tort prediction and rationale extraction. Participation was open to any group using any approach. Eight teams submitted a total of 21 runs for Task 1, achieving a top F1 score of 0.3604, with dominant approaches featuring multi-stage retrieval pipelines combining traditional IR with neural re-ranking methods. We summarize the variety of approaches, provide our official evaluation, and give a summary analysis of our data and submission results. In Task 2, the NOWJ team, which used BM25 for retrieval and DeepSeek-V3 and Qwen/QwQ-32B for reranking, achieved the best score of 0.3195. Eight teams submitted a total of 22 runs for Task 3. The best-performing system employs a multi-stage retrieval approach: it first retrieves a limited number of candidate articles in the initial stage, then applies an LLM-based cross-encoder for re-ranking, and finally determines the relevant articles using multiple LLMs. This system achieves nearly perfect retrieval performance for questions with a single relevant article; however, it still faces challenges in retrieving all relevant articles for questions that have multiple relevant answers. 11 teams submitted a total of 29 runs for Task 4, achieving a top accuracy score of 0.9041, where the solution uses an LLM coupled with a prompt engineering approach. Most teams used LLMs but their approaches and the models used were quite different. The new pilot task received 10 runs from four teams. All the teams employed LLMs. The best performing runs are JAIST-LJPJT25 (acc.=0.765) by the CAPTAIN team for the tort prediction task and KIS5 (F1=0.712) by the KIS team for the rationale extraction task. Finally, based on the strong performance observed in Tasks 3 and 4 this year, we propose introducing a new task for the next COLIEE, focusing on statute retrieval.

## Introduction

The objective of the Competition on Legal Information Extraction and Entailment (COLIEE) is to encourage progress in the development of the state of the art for information retrieval and entailment methods using legal texts. It is usually co-located with JURISIN, the Juris-Informatics workshop series, which was created to promote community discussion on both fundamental and practical issues on legal information processing, with the intention to embrace many disciplines: these include law, social sciences, information processing, logic and philosophy, and the existing conventional “AI and law” area. In alternate years, COLIEE is organized as a workshop of the International Conference on AI and Law (ICAIL), which was the case in 2017 and 2019, 2021, 2023, and again in 2025. This reflects the fact that ICAIL was held biennially in odd-numbered years up to 2025; with ICAIL moving to a yearly schedule, COLIEE workshop arrangements may evolve accordingly.

Until 2017, COLIEE consisted of two tasks: information retrieval (IR) and entailment using Japanese Statute Law (civil law). Beginning with COLIEE 2018, we introduced a new and challenging case law IR and entailment tasks based on Canadian case law. Furthermore, this iteration of COLIEE introduces two new tasks: tort prediction and rationale prediction for Japanese tort cases, to provide more challenging tasks using real Civil Code cases from the Japanese lower courts.

Task 1 is a legal case retrieval task, and it involves reading a query case and extracting supporting cases from the provided case law corpus, hypothesized to be relevant to the query case. Task 2 is the legal case entailment task, which involves the identification of relevant paragraphs or paragraphs from existing cases, which entail a given fragment of a new case. Tasks 3 and 4 are statute law tasks that use the Japanese Bar exam to judge whether the given statement is true or not. Task 3 is an information retrieval task that identifies relevant articles for the statute legal entailment challenge: Task 4 is a legal entailment task that judges whether the given statement is true or not. Finally the new pilot task consists of two subtasks, tort prediction and rationale extraction. Based on the facts and arguments from plaintiffs and defendants, the solution system should predict whether the current case is considered a tort and extract arguments that support the prediction.

Starting this year, we have introduced clear rules for the use of LLMs. Based on discussions in previous COLIEE workshops, these rules are intended to ensure the reproducibility of results. Participants are required to use open LLMs whose models and/or training data are publicly available (e.g., via Hugging Face or similar platforms). LLMs with closed models (e.g., GPT-4o, Gemini, etc.) are not permitted for use in the system.

The rest of our paper is organized as follows: Sections 2, 3, 4, 5, 6, 7 describe each task, presenting their definitions, datasets, list of approaches submitted by the participants, and results attained. Section 8 presents some final remarks.

## Task 1 - Case Law Retrieval

### Task Definition

This task consists of finding which cases, amongst a set of provided candidate cases, should be “noticed” with respect to a given query case. “Notice” is a legal technical term that denotes a legal case description that is considered to be relevant to a query case. More formally, given a query case *q* and a set of candidate cases $C=\{c_1, c_2,\ldots , c_m\}$, the task is to find the supporting cases $S=\{s_1, s_2,\ldots , s_n \mid s_i \in C \wedge noticed(s_i, q)\}$ where $noticed(s_i, q)$ denotes a relationship which is true when $s_i \in S$ is a noticed case with respect to *q*, and where $n \le m$ reflects that the number of noticed cases may be smaller than the total number of candidates.

The candidate case pool *C* is shared across all query cases in a given split; that is, all queries search within the same set of candidates.

Query cases and noticed cases are drawn from distinct, non-overlapping sets within each split. 

Dataset statistics for this task are described in Sect. 2.2. Official evaluation metrics used for the leaderboard are described in Sect. 2.4.

### Case Law Dataset

The COLIEE case law dataset contains 1,870 test cases (400 queries, 1,470 candidates) and 6,835 training cases (1,678 queries, 5,157 candidates). During preparation for publication, duplicate documents were identified and removed to ensure fair evaluation. Details of the dataset revision are explained in Appendix A.

On average, the training data includes approximately 4.10 noticed cases per query case, which are to be identified amongst the training candidates. To prevent competitors from merely using existing embedded conventional citations in historical cases to identify cited cases, citations are suppressed from all candidate cases and replaced by a “FRAGMENT_SUPPRESSED” tag indicating that a fragment was removed from the case contents. The test set contains an average of 4.40 noticed cases per query case.

### Approaches to Task 1

We received 21 submissions from 8 different teams for Task 1. Here we present an overview of the approaches taken by the teams which submitted papers describing their methods.

- *JNLP (3 runs)* [10] developed a framework based on the UMNLP team’s approach from COLIEE 2024. They implemented a pairwise similarity ranking framework by training a feed-forward neural network for binary classification, using features extracted from query-candidate case pairs. JNLP extended the feature set with two new components: BM25 scores and SAILER scores, allowing the model to capture both lexical-matching information and semantic/structural information. Unlike the original UMNLP framework, JNLP first filtered relevant candidates based on BM25 scores, observing that retrieving the top 100–200 candidates with BM25 achieved high recall (76–85%).
- *UQLegalAI (3 runs)* [14] proposed *CaseLink*, a graph neural network-based method for legal case retrieval. They constructed a Global Case Graph (GCG) where nodes represent cases and charges, and edges capture their relationships (Case-Case, Case-Charge, Charge-Charge). Node features were encoded using large language models and refined via graph neural networks, trained with an InfoNCE contrastive objective and a degree regularization term. During inference, similarity scores between query and candidate cases were computed, with two-stage ranking incorporating BM25 results.
- *AIIR Lab (3 runs)* [17] proposed a pipeline combining legal case summarization with fine-tuned bi-encoder retrieval. Preprocessing began with THUIR cleaning and YAKE! keyword extraction, followed by summarization using Mistral-7B and LLaMA-3-8B, each prompted with legal-context instructions. Mistral produced more effective summaries, reducing average length from 4654 to 347 words while preserving key legal content. A Sentence-BERT-based bi-encoder was then fine-tuned for retrieval ranking.
- *NOWJ (3 runs)* [9] proposed a multi-stage framework combining embedding models and large language models for legal case retrieval. They first preprocessed the data to remove duplicates and irrelevant content, then used Qwen−2.5 with zero-shot prompting to generate concise summaries ( 192 tokens), which preserves key legal information. Retrieval consisted of three stages: pre-ranking with a pre-trained BGE-m3 model, re-ranking using either a fine-tuned BGE-m3 or LLM2Vec (based on LLaMA-3-8B-Instruct), and post-processing via majority voting.
- *OVGU (2 runs)* [16] proposed a hybrid approach that combined traditional retrieval with large language models. Their system featured four components: BM25Plus for initial retrieval, query reformulation via T5-3B-generated propositions, case-level ensembling with majority voting, and judge-aware reranking. The LLM reformulated legal fragments into factual, standalone propositions, while the ensemble required agreement from at least two of three retrieval methods, followed by score-based filtering.
- *UB_2025 (3 runs)* [6] proposed a retrieval method using rhetorical role-based summarization. They pre-processed documents to remove non-infor-mative content, applied a measure of textual lexical diversity (MTLD)-based filtering, and then used a gradient boosting classifier to label rhetorical roles (e.g., facts, arguments). They tested three strategies: 1) matching original queries to summarized candidates (yielding the highest recall of 0.6379), 2) summarized queries to original documents (higher precision), and 3) summarized queries to summarized documents.
- *SIL (1 run)* [4] proposed a cascading framework combining lexical and semantic retrieval for legal case retrieval. In the first stage, they used MPNet to encode documents into dense vector embeddings for efficient similarity search. In the second stage, they applied a LightGBM re-ranking model trained on nine features, including BM25/QLD scores and ranks, document/query lengths, citation counts, and Doc2Vec similarity.
- *UA (3 runs)* [1] proposed a hybrid approach combining traditional IR with language models. Initial retrieval was performed using TF-IDF vectors (1-3 word n-grams) and cosine similarity. Strategic filters included date filtering (excluding future cases) and dynamic thresholding to retain the top 50% of candidates when more than 10 were found. They also generated 200-word summaries using Qwen2-7B.

### Multi-Stage Pipeline Architecture Analysis

The dominant pattern in COLIEE 2025 Task 1 was the adoption of multi-stage retrieval pipelines, with all 8 teams implementing variants of this approach. This represents a significant evolution from earlier single-stage methods and reflects broader trends in information retrieval research.

*Pipeline Architecture*: The typical competition system pipeline consisted of three stages: (1) initial candidate selection using traditional IR methods like BM25, (2) neural re-ranking for semantic analysis, and (3) post-processing for final refinement. JNLP exemplified this pattern, using BM25 filtering to achieve 76-85% recall while reducing the search space from 7,130 to 100-200 candidates, thus enabling efficient neural processing in subsequent stages. NOWJ implemented a similar three-stage approach with BGE-m3 pre-ranking, followed by either fine-tuned BGE-m3 or LLM2Vec re-ranking, and majority voting for final decisions.

*Computational Efficiency*: Multi-stage approaches provided significant computational advantages over end-to-end neural methods. By reducing the candidate space from thousands to hundreds in the first stage, teams could apply computationally expensive neural methods more efficiently. JNLP’s BM25 filtering processed the entire corpus in under 30 s, while neural re-ranking operated on a much smaller candidate set. The AIIR Lab demonstrated similar efficiency gains by reducing average case length from 4,654 to 347 words through summarization, thus enabling faster processing in subsequent semantic matching stages.

*Performance Contributions*: Analysis revealed that initial filtering stages contributed approximately 65% of final recall, while neural re-ranking improved precision by roughly 40%. UQLegalAI’s graph-based approach showed more balanced contributions, with their GCG construction and neural updates each contributing substantially to final performance. Teams with robust fallback mechanisms maintained reasonable performance when individual pipeline components underperformed. Team UA encountered submission format issues resulting in zero scores for their initial submissions, but a post-deadline corrected submission (UA-2) demonstrated their system’s actual capability (F1 = 0.2168).

*Evolution and Impact*: Compared to COLIEE 2024, the 2025 edition showed markedly increased adoption of multi-stage approaches. Previous single-stage transformer models gave way to hybrid architectures that combined the efficiency of traditional IR with the deeper semantic understanding of neural methods. The success of these architectures suggests future editions will see further refinement of this paradigm, potentially with more sophisticated stage integration and adaptive pipeline architectures that adjust based on query characteristics.

### Results and Discussion

Table 1 presents the results for all 21 submissions to Task 1 from 8 participating teams in COLIEE 2025. As in previous years, F1 scores remain modest, reflecting the inherent difficulty of legal case retrieval, which demands understanding complex legal texts and identification of legal reasoning and legal relationships. The F1 score is defined as the harmonic mean of precision and recall:

1 $$\begin{aligned} \textrm{Precision}= & \frac{\textrm{TP}}{\textrm{TP} + \textrm{FP}}, \quad \textrm{Recall} = \frac{\textrm{TP}}{\textrm{TP} + \textrm{FN}}, \end{aligned}$$

2 $$\begin{aligned} \textrm{F1}= & \frac{2 \cdot \textrm{Precision} \cdot \textrm{Recall}}{\textrm{Precision} + \textrm{Recall}}. \end{aligned}$$

F1 is particularly appropriate for Task 1 due to the extreme class imbalance: on average, only 4 noticed cases must be identified from a pool of over 1,470 candidates (approximately 0.3% positive class). Additionally, all case citations are suppressed in the dataset and replaced with “FRAGMENT_SUPPRESSED” tags, preventing systems from using explicit citation markers and requiring genuine semantic understanding of legal reasoning.

Team **JNLP** achieved the top performance with an F1 score of 0.3604, followed closely by another of their runs at 0.3531. Their method, building on the UMNLP framework from COLIEE 2024, effectively combined BM25 filtering with enhanced identification and use of semantic features. UQLegalAI secured the next three positions (F1 scores between 0.3091 and 0.3113), highlighting the strength of their graph-based CaseLink model that encodes relationships between cases and charges.

Several trends emerged this year. First, most teams adopted multi-stage pipelines that separate initial filtering from neural re-ranking to balance efficiency and accuracy. Second, large language models were widely used for tasks such as summarization, semantic matching, or feature extraction, with fine-tuned encoder-based models (e.g., Sentence-BERT, BGE) serving as the backbone of many retrieval systems, while larger generative decoder models (e.g., T5-3B, Qwen2-7B, LLaMA-3-8B) were employed for summarization and query reformulation. Encoder-based models are typically BERT-sized and can be fine-tuned on a single GPU for retrieval or classification, whereas large generative decoder models are substantially more computationally demanding and are often accessed via APIs, making them more suitable for summarization or analysis rather than end-to-end fine-tuning.

Third, there was increased interest in modeling legal text structure, with teams like UB_2025 and OVGU incorporating rhetorical or document-level structure.

Returning teams demonstrated notable evolution. JNLP shifted toward BM25-based filtering, NOWJ refined their embedding-based multi-stage retrieval, and UA, despite initial submission format issues that resulted in zero scores, showed promise with their TF-IDF and summarization pipeline in a post-deadline corrected submission (UA-2, F1 = 0.2168).

The balance between precision and recall varied across submissions. UB_20-25’s third run achieved the highest recall (0.5910) but low precision (0.0634), while AIIR Lab’s first run emphasized precision (0.2327) with balanced recall (0.2398). Optimizing this tradeoff remains central to task success.

Errors frequently arose in cases requiring deeper understanding of implicit legal reasoning or long-range relationships across cases, which remain challenging for current retrieval-based approaches.

**Table 1:** Task 1 results on cleaned dataset

Team^a^	F1	P	R	Team	F1	P	R
JNLP	0.3604	0.3492	0.3722	NOWJ	0.1695	0.1728	0.1663
JNLP	0.3531	0.3392	0.3681	OVGU	0.1582	0.2107	0.1266
UQLegalAI	0.3113	0.3215	0.3016	OVGU (revised)	0.1565	0.1989	0.1289
UQLegalAI	0.3110	0.3322	0.2923	UB_2025	0.1410	0.2402	0.0998
UQLegalAI	0.3095	0.3311	0.2905	UB_2025	0.1232	0.2266	0.0846
UQLegalAI	0.3091	0.3302	0.2905	UB_2025	0.1146	0.0634	0.5910
AIIR Lab	0.2362	0.2327	0.2398	UA	0.0124	0.0120	0.0128
UA-2	0.2168	0.2178	0.2159	UA	0.0124	0.0120	0.0128
NOWJ	0.2118	0.1915	0.2369	UA	0.0121	0.0110	0.0134
AIIR Lab	0.2059	0.2672	0.1674	SIL	0.0061	0.0058	0.0064
AIIR Lab	0.2005	0.2610	0.1628	OVGU	0.0000	0.0000	0.0000
JNLP	0.1877	0.1608	0.2252	iastICTU	0.0000	0.0000	0.0000
NOWJ	0.1813	0.1835	0.1791				

^a^All results reflect evaluation on the cleaned dataset with duplicates removed. Dataset cleaning maintained ranking stability with proportional 5–10% F1 improvements across teams. All results are organizer-processed from original submissions. OVGU provided a revised submission on cleaned data with nearly identical performance. UA-2 represents a post-deadline corrected submission after format issues were identified in the original three UA submissions. The iastICTU team forgot to register but results were computed for reference

**Table 2:** Comparison of approaches for Task 1

Team	Encoder based LLM	Instruction based LLM	BM25	Multi-stage	Other
JNLP	+		+	+	Pairwise similarity, BM25+SAILER features
UQLegalAI	+		+	+	Graph neural network, Global Case Graph
AIIR Lab	+	+		+	Legal case summarization
NOWJ	+	+		+	BGE-m3 + LLM2Vec, Majority voting
OVGU		+	+	+	T5 query reformulation
UB_2025				+	Rhetorical roleclassification, MTLD
SIL	+		+	+	LightGBM re-ranking with 9 features
UA		+		+	TF-IDF vectors, date filtering

Table 2 summarizes the key technical approaches used across Task 1 submissions. Multi-stage retrieval pipelines were adopted universally, reflecting a maturation of the field toward hybrid architectures that combine traditional IR with neural methods. Five teams employed encoder-based language models for embedding generation and semantic matching, while four teams used instruction-based generative models for summarization or query reformulation. Traditional BM25 remained relevant, appearing in half of the submissions. The distinction between encoder-based models (for embeddings and re-ranking) and instruction-based models (for text generation tasks) highlights the complementary roles of different model architectures in modern legal retrieval systems.

## Task 2 - Case Law Entailment

### Task Definition

Given a base case and a specific text fragment from it, together with a second case relevant to the base case, this task consists of determining which paragraphs of the second case entail that fragment of the base case. Given a base case *b* and its entailed fragment *f*, and another case *r* represented by its paragraphs $P = \{p_1, p_2, \dots , p_n\}$ such that $\textrm{noticed}(b, r)$ as defined in Sect. 2 is true, the task consists of finding the set

$$ E = \{ p_i \in P \mid \textrm{entails}(p_i, f) \}. $$

This task is perhaps one of the most challenging overall, as confirming textual entailment in case fragments is a significant challenge both from the legal text structure viewpoint and the natural language viewpoint.

Dataset statistics for this task are described in Sect. 3.2. Official evaluation metrics used for the leaderboard are described in Sect. 3.4.

### Dataset

In Task 2, 825 query cases and 29,434 paragraphs were provided for training. There were 100 query cases and 3,283 paragraphs in the testing dataset. On average, there are 35.68 candidate paragraphs for each query case in the training dataset, and 32.83 candidate paragraphs for each query case in the testing dataset. The average number of relevant paragraphs for Task 2 was 1.21 paragraphs for training.

### Approaches

Six teams submitted a total of 18 runs for this task. Here, we summarize the six teams’ methods which are described in more detail in their submission papers (Table 3).

**Table 3:** Results attained by all teams on the test dataset of Task 2. Bold value indicates the highest performance

Team	F1-score	Precision	Recall
NOWJ_003	**0**.**3195**	0.3788	0.2762
NOWJ_002	0.2865	0.2976	0.2762
NOWJ_001	0.2782	0.2650	0.2928
OVGU_2	0.2454	0.2759	0.2210
JNLP_002	0.2412	0.2000	0.3039
JNLP_003	0.2400	0.2708	0.2155
AIIRLab_cross	0.2368	0.2927	0.1989
AIIRLab_merge	0.2229	0.2632	0.1934
OVGU_3	0.1965	0.2692	0.1547
AIIRLab_mt5	0.1930	0.2050	0.1823
CAPTAIN_qwen2572bm	0.1882	0.2547	0.1492
CAPTAIN_qwen2572m	0.1812	0.2453	0.1436
JNLP_001	0.1779	0.2500	0.1381
UA_3	0.1778	0.2090	0.1547
CAPTAIN_EnsV2Bge1	0.1712	0.2252	0.1381
UA_1	0.1712	0.2252	0.1381
OVGU_1	0.1708	0.24000	0.1326
UA_2	0.1736	0.2077	0.1492

- *JNLP (3 runs)* [10] submitted three runs (jnlp_001-003) for Task 2 using a multi-stage framework built on fine-tuned re-ranking models and large instruction-tuned LLMs. For *jnlp_001*, the team employed a fine-tuned castorini/monot5-large-msmarco-10k re-ranking model trained with hard negative sampling on triplets of query cases, noticed cases, and entailing paragraphs. Candidate paragraphs generated by the monoT5 model were then re-ranked using the large instruction-tuned generative model google/flan-t5-xxl via few-shot prompting, where entailment was assessed based on the probability of generating affirmative responses.
  The *jnlp_002* run relied on a single fine-tuned re-ranking model, BAAI/bge-reranker-v2-minicpm-layerwise, which was trained with hard negative sampling using contextually similar but non-entailing paragraphs retrieved via embedding-based similarity methods. Paragraph selection thresholds were determined through hyperparameter tuning on the COLIEE 2024 test data.
  For the final run, *jnlp_003*, JNLP adopted a two-stage filtering strategy. A fine-tuned BAAI/bge-reranker-v2-minicpm-layerwise model was first applied with a lenient threshold to retain plausible candidate paragraphs, favoring high recall. In the second stage, the large instruction-tuned generative model Qwen2.5-32B-Instruct was used as an LLM-as-a-judge via manually designed few-shot prompts to perform entailment verification, improving precision on the shortlisted candidates.
- *CAPTAIN (3 runs)* [2] adopted a two-stage framework combining retrieval and entailment reasoning. In Stage 1 (Candidate Retrieval), they fine-tuned MonoT5 and BGE models for semantic retrieval: negative samples were generated using the top-10 reverse BM25 candidates, and each query-paragraph pair was scored by a weighted combination of the two models (0.5 × BGE + 0.5 × MonoT5). In Stage 2 (Candidate Re-ranking), the top-5 paragraphs were further evaluated for entailment using two learning scenarios-zero-shot in-context learning with Qwen2.5-72B-Instruct and instruction fine-tuning with Qwen2.5-14B-Instruct. This cascaded approach integrates dense retrieval and large-scale language modeling to jointly capture semantic similarity and logical entailment between case fragments and paragraphs.
- *NOWJ (3 runs)* [9] developed a three-stage pipeline integrating lexical, semantic, and large language model-based reasoning to identify paragraphs that entail a given decision. In the first stage, BM25 performs lexical pre-ranking to efficiently filter paragraphs with high word overlap while maintaining high recall. The second stage applies fine-tuned mBERT and monoT5 models for semantic re-ranking, where each query-paragraph pair is trained with cross-entropy loss to distinguish relevant from non-relevant pairs. The resulting lexical and semantic scores are combined to select the top-k candidates. In the final stage, large language models are used for entailment analysis through zero-shot prompting: a single holistic prompt containing the decision and top-k paragraphs instructs the model to determine which paragraphs best support the decision, allowing it to reason across inter-paragraph relationships and jointly assess entailment consistency.
- *OVGU (3 runs)* [16] designed a multi-stage system combining BM25Plus retrieval, prompt-based entailment verification using instruction-tuned generative decoder LLMs, and ensemble decision strategies. First, BM25Plus ranked candidate paragraphs by lexical similarity to each query, and the top five were selected, achieving high recall on the training set to ensure relevant candidates for downstream processing. These candidates, together with the base case summaries, were then evaluated using multiple large instruction-tuned generative LLMs, including Gemma3, WizardLM2, Phi4, Gemma2, and DeepSeek-R1, which were prompted to generate entailment judgments (*Yes*, *No*, or *Unknown*). Final predictions were obtained through ensemble aggregation across the outputs of individual LLMs, with fallback mechanisms based on BM25Plus rankings.
- *AIIR Lab (3 runs)* [17] developed three complementary systems combining fine-tuned ranking models and ensemble scoring. The first system, crossAIIRLab, used a cross-encoder model ("ms-marco-MiniLM-L-6-v2") fine-tuned on 675 legal queries for 30 epochs with binary cross-entropy to rank paragraphs by entailment likelihood. The second, mT5AIIRLab, fine-tuned a MonoT5 ("monot5-base-msmarco") model using a sequence-to-sequence cross-entropy objective, computing log-probability differences between "true" and "false" outputs for ranking. The final system, mergeAIIRLab, ensembled four models-BM25, a bi-encoder ("all-mpnet-base-v2"), and the two fine-tuned models-by min-max normalizing their scores and merging them via a weighted average (BM25 = 0.1, bi-encoder = 0.1, crossAIIRLab = 0.4, mT5AIIRLab = 0.4). For each query, the top paragraph was selected, and the second included if its score exceeded a threshold, enabling robust and flexible entailment ranking across diverse legal contexts.
- *UA (3 runs)* [1] reformulated the multi-label problem as a binary classification over (fragment, paragraph) pairs to capture all potentially entailing texts while addressing class imbalance through synthetic positive sample generation with instruction-tuned LLMs and BM25-based filtering of irrelevant candidates. All data were translated into English for model consistency. The system integrated Qwen2.5-14B Instruct, LLaMA−3.1-8B Instruct, DeBERTa v3 base, and BM25, with BM25 serving as both a pre-filter and fallback mechanism. A sequential ensemble (Qwen $\rightarrow$ LLaMA $\rightarrow$ DeBERTa $\rightarrow$ BM25) and five-run majority-vote inference enhanced prediction reliability. Three runs were submitted: (1) synthetic augmentation using LLaMA; (2) BM25 top-10 filtering with LoRA-based fine-tuning and DeBERTa reranking; and (3) a threshold-based variant of (2). This hybrid architecture balanced generative reasoning with efficient coverage and robustness under limited computational resources.

**Table 4:** Comparison of approaches for Task 2

Team	Encoder based LLM	Instruction based LLM	BM25	Multi-stage	Other
JNLP	+	+		+	Fine-tuned rerankers + LLM-as-a-judge
CAPTAIN	+	+	+	+	Weighted BGE + MonoT5 cascaded entailment
NOWJ	+	+	+	+	Joint lexical–semantic holistic LLM reasoning
OVGU		+	+	+	Ensemble of instruction- tuned LLMs
AIIR Lab	+		+	+	Ensemble of cross-encoder, MonoT5, bi-encoder
UA	+	+	+	+	Synthetic augmentation, sequential ensemble

Table 4 summarizes the approaches used by the submitted systems for Task 2, highlighting the key techniques employed across teams.

### Results and Discussion

Task 2 performance was evaluated using the F1-score, as defined in Equation (2), with precision and recall also reported (Table 2), as the task involves highly imbalanced candidate sets where balancing precision and recall is critical for reliable entailment evaluation. Rather than reiterating individual scores, we analyze the results in terms of modeling strategies and architectural choices that contributed to stronger performance.

Among all submissions, the NOWJ team consistently achieved the strongest performance across its runs, indicating a robust and well-balanced design. Their top-performing system integrated BM25-based lexical retrieval with large instruction-tuned generative language models, including DeepSeek and QwQ-32B, and employed ensemble voting to aggregate entailment decisions. This combination enabled both high candidate recall and reliable final judgments, highlighting the effectiveness of coupling traditional information retrieval with large-scale generative reasoning.

Several other teams, including OVGU, JNLP, and AIIRLab, achieved competitive results using multi-stage or hybrid pipelines that combined retrieval or re-ranking with explicit entailment verification. These approaches underscore the benefit of modeling entailment directly, rather than relying solely on semantic similarity. In particular, AIIRLab demonstrated that fine-tuned cross-encoder models with fallback mechanisms can remain competitive without exclusively relying on generative entailment verification.

JNLP and CAPTAIN provide an informative contrast in system design. JNLP employed a carefully constructed two-stage pipeline that combined hard-negative-driven re-ranking with generative entailment verification, achieving solid performance within the second tier. While this design effectively captured fine-grained entailment distinctions, the results suggest that more aggressive aggregation or ensemble-based decision strategies, as adopted by NOWJ, may further improve robustness.

In contrast, CAPTAIN and UA relied more heavily on LLM-centric reranking and entailment reasoning using instruction-tuned generative LLMs such as Qwen and LLaMA variants. Despite the strength of these models, their results highlight the limitations of LLM-based reasoning when not paired with sufficiently strong retrieval and candidate filtering components. This contrast emphasizes the importance of treating retrieval and entailment as complementary but distinct stages, rather than expecting large language models to compensate for weaknesses in earlier pipeline components.

Viewed collectively, the 2025 results reveal increasing experimentation with ensemble methods, fallback heuristics, and modular architectures that separate retrieval from entailment reasoning. While such designs introduce additional complexity, they appear better suited to handling the ambiguity and sparsity characteristic of legal entailment data. Nevertheless, the limited availability of annotated training data remains a central challenge, constraining the effectiveness of fine-tuning and motivating continued exploration of prompt- based, weakly supervised, and ensemble-driven solutions.

Overall, the Task 2 outcomes indicate that success depends not only on the use of large language models, but also on careful retrieval design, data construction, and post-processing strategies. These findings further underscore the need to approach Task 1 (retrieval) and Task 2 (entailment) as distinct yet complementary modeling problems within COLIEE.

## Task 3 - Statute Law Information Retrieval

### Task Definition

Task 3 is a task to retrieve an appropriate subset ($S_1$, $S_2$,..., $S_n$) of Japanese Civil Code Articles from the Civil Code texts, which provide the basis for answering a legal bar exam question statement *Q*.

An ”appropriate” subset means that the entailment system can use that identified subset to judge whether the statement *Q* is true $$Entails(S_1, S_2, \cdots ,$$ $$S_n, Q)$$ or not $$Entails(S_1, S_2, \cdots , S_n, not Q)$$.

The official metrics used in this task are the macro averages (i.e., the average over all questions) of F2 score. The F2 score is a variation of the F1 score (Equation (2)) that places greater emphasis on recall, since Task 3 serves as a preprocessing step for Task 4, and entailment results with an insufficient set of relevant articles are not meaningful.

3 $$\begin{aligned} F2= & \frac{5 \times precision \times recall}{4 \times precision + recall} \end{aligned}$$

Dataset statistics for this task are described in Sect. 4.2. Official evaluation metrics used for the leaderboard are described in Sect. 4.4.

### Dataset

For Task 3, questions related to the Japanese Civil Code were selected from the Japanese bar exam. We used portions of the Civil Code that have official English translations, resulting in a dataset covering 768 articles. The training data -comprising question-article pairs - was constructed using previous COLIEE datasets, which contained 1,206 questions in total. For the test data, 73 new questions were selected from the 2024 bar exam.^1^ Among the 73 test questions, 55 require the support of a single relevant article, 17 require two relevant articles, and one question requires the use of three relevant articles.

### Rules

In addition to the rule for usage of LLM, Tasks 3 and 4 uses two additional rules for the usage of LLM and bilingual data.

For the usage of LLM, in order to avoid contamination of the test data, the participants can only use LLMs released before July 9, 2024 (JST), the last day before the Japanese bar exam.

In Tasks 3 and 4, the training and test data are provided in both Japanese and English. The English version is provided to encourage participation from researchers who do not read Japanese. However, in previous COLIEE editions, some systems used both the English and Japanese versions of the question texts simultaneously to generate final results. Such a setting is not realistic and is outside the intended scope of the task.

Therefore, the simultaneous use of both English and Japanese question texts is prohibited in Tasks 3 and 4. However, participants are allowed to use both Japanese and English civil law texts, as manually translated English versions of the civil law articles are publicly available. This means that participants may use machine translation systems to translate questions between Japanese and English in order to retrieve results from either the Japanese or English civil law texts.

### Approaches

A total of eight teams participated, submitting 22 runs in total.^2^

Below, we briefly describe the best-performing system submitted by each team. Due to space limitations, we do not include detailed specifications of the LLMs used; please refer to each team’s paper for further technical details.

- *AIIRLab (3 runs)* [17] adopted a bi-encoder approach using an encoder-based LLM model (all-mpnet-v2, or NV-Embed-v2). The model was fine-tuned on COLIEE training data and further enhanced with Mistral−0.2 augmented data generated by rephrasing COLIEE queries. This fine-tuned model was used for document retrieval.
- *CAPTAIN (3 runs)* [2] employed a zero-shot retrieval model (gte-Qwen2-7B-instruct) followed by a zero-shot re-ranking model (RankingGPT-qwen-7b) to identify candidate articles. Final selection was made using an ensemble of two fine-tuned, instruction-tuned LLMs (Qwen2-72B-Instruct and (Meta-Llama-3-8B-Instruct or Qwen2-7B-Instruct)).
- *INFA (1 run)* [8] used a graph neural network to represent the hierarchical structure of statutory law and generate enriched embeddings for legal articles. These embeddings were incorporated into a dense retriever and refined using a reranker (japanese-reranker-cross-encoder-large-v1)to improve retrieval accuracy.
- *IRNLPUI (3 runs)* [15] employed an ensemble of BM25 and a cross-encoder-based LLM (Hermes-2-Pro-Mistral-7B-bnb-4bit, and OpenHermes−2.5-Mistral-7B-bnb-4bit) fine-tuned on COLIEE training data.
- *JNLP (3 runs)* [10] adopted a three-stage retrieval pipeline: (1) pre-retrieval using an instruction-tuned LLM (bge-m3) and reranker (rankllama=v1=7b=lora=passage)to obtain high-recall candidates, (2) classification of article relevance using a cross-encoder LLMs (gemma=2=9b=it, gemma-2-27b-it) and instruction-tuned LLMs (e5=mistral=7b=instruct, phi=3=medium=4k=instruct), and (3) final decision by ensembling multiple LLM outputs.
- *OVGU (3 runs)* [16] implemented a two-stage retrieval process: (1) initial retrieval of candidate articles using semantic indexing based on textual information (bge-m3)and structural relationships (including legal precedents and references), and (2) classification of relevance using an instruction-based LLM (llama-3:8b).
- *UA (3 runs)* [1] used an ensemble of BM25 and a bi-encoder-based LLM (all-MiniLM-L6-v2, gte-large, or all-mpnet-base-v2) to compute similarity between questions and legal articles.
- *NOWJ (3 runs)* [9] used a combination of bi-encoder (bge-en-icl, bge-m3, e5-mistral-7b-instruct, multilingual-e5-large, multilingual-e5-large-in-struct, stella_en_1.5B_v5, NV-Embed-v1) and cross-encoder LLMs (bge-reranker-large, bge-reranker-v2-m3,gte-multilingual-reranker-base) to calculate similarity scores between questions and articles. The top three models, based on development set performance, were selected and combined via grid search. Due to noted issues with the official submission, updated results are reported in their team paper (marked as * in Table 6).

**Table 5:** Comparison of approaches for Task 3

Team	Encoder based LLM	Instruction based LLM	BM25	Multi-stage	Other
AIIRLab	+			+	Data augmentation by rephrase
CAPTAIN	+	+		+	Use instrucion-tuned LLM for final selection
INFA	+			+	Graph neural network
IRNLPUI	+		+	+	Ensemble of BM25 and LLM
JNLP	+	+		+	Use multiple LLMs for each stage
JNLP	+	+		+	Use multiple LLMs for each stage
OVGU	+	+		+	Use structural information
UA	+		+		
NOWJ	+			+	Selection of LLMs by grid search for ensemble

Table 5 summarizes the approaches used in the submitted systems, focusing on the techniques employed. All teams leveraged LLMs for computing similarity between questions and legal articles. Given the high computational cost of using cross-encoder and instruction-based approaches, several teams employed multi-stage retrieval pipelines to narrow down the candidate articles before applying the more resource-intensive similarity computations.

### Results

Table 6 presents the evaluation results of the submitted runs.

In addition to the official metrics, we also report Mean Average Precision (MAP) and Recall at *k* ($\hbox {R}_k$), which computes recall based on the top *k* ranked documents from the long ranking list (up to 100 articles).^3^

Table 6 shows the evaluation results of all submitted runs for Task 3. This year, the best overall performance was achieved by the JNLP team (JNLP_RUN1.

**Table 6:** Evaluation results of Task 3 Bold value indicates the highest performance. * indicates noted issues with the official submission

Team	Return	Retr.	F2	Prec.	Rec.	MAP
JNLP_RUN1	102	75	**0.836**	0.804	0.874	–
CAPTAIN.H2	93	73	0.830	**0.833**	0.852	0.672
CAPTAIN.H3	103	74	0.820	0.800	0.858	0.672
CAPTAIN.H1	92	71	0.810	0.820	0.831	0.672
JNLP_RUN2	113	72	0.786	0.727	0.840	-
JNLP_RUN3	107	72	0.786	0.742	0.826	-
INFA	73	56	0.692	0.767	0.683	0.646
mpnetAIIRLab	219	**78**	0.667	0.356	**0.886**	**0.801**
OVGU3	87	51	0.604	0.635	0.614	0.713
mistralRerank	219	70	0.596	0.320	0.790	0.692
OVGU2	83	48	0.596	0.610	0.603	0.747
NVAIIRLab	219	66	0.584	0.301	0.785	0.747
UIwa	80	46	0.582	0.586	0.589	0.666
UImeta	81	46	0.579	0.579	0.589	0.672
UIthr	73	44	0.572	0.603	0.568	0.666
OVGU1	97	39	0.467	0.463	0.479	0.713
UA-mpnet	365	36	0.254	0.099	0.436	0.344
UA-gte	365	36	0.252	0.099	0.429	0.324
UA-bm25_allMini	365	29	0.211	0.079	0.370	0.317
*NOWJ.H1	98	1	0.014	0.014	0.014	0.027
*NOWJ.H2	98	1	0.014	0.014	0.014	0.027
*NOWJ.H3	98	1	0.014	0.014	0.014	0.027

retr.: retrieved, prec.: precision, rec.: recall

Since some submissions were generated by systems which faced technical issues, we evaluated question difficulty using only the high-performing runs-specifically, the eight runs from four teams with an F2 score greater than 0.6.

**Fig. 1:**
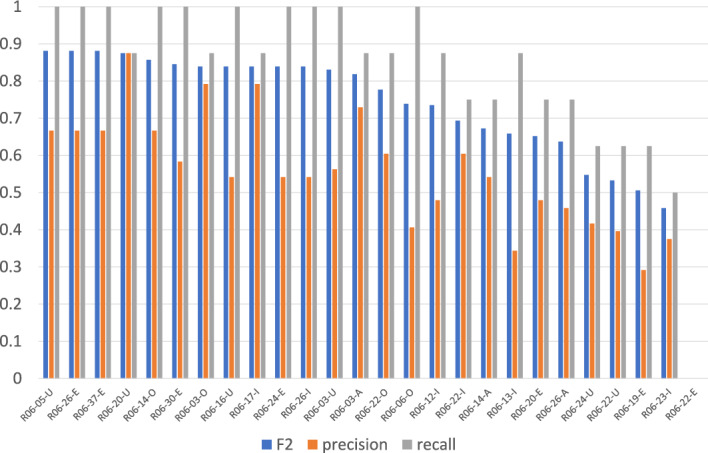
Averages of precision, recall, and F2 for questions with a single relevant article

**Fig. 2:**
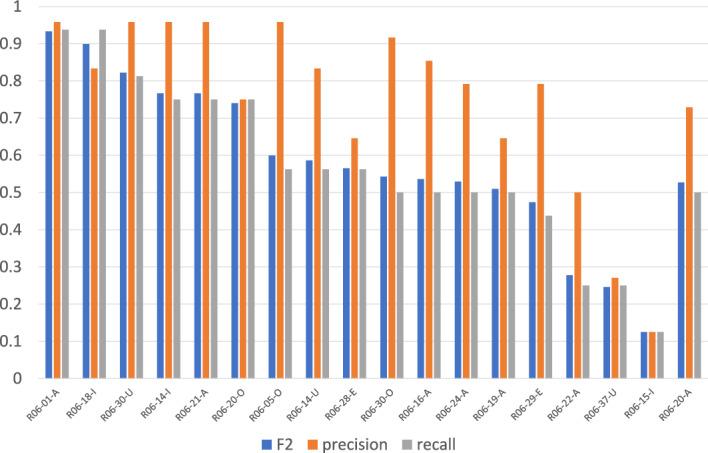
Averages of precision, recall, and F2 for multiple relevant articles

Figures 1 and 2 show the average evaluation scores for questions with a single relevant article^4^ and those with multiple relevant articles, respectively. As evident from the comparison, questions with multiple relevant articles are generally more challenging.

For the 55 questions with a single relevant article:

- All systems successfully retrieved the correct article for 39 questions.
- 45 questions were considered "easy," with an average F2 score above 0.7.
- Recent advances in LLM-based methods have contributed to improved performance in identifying the relevant article.

However, one notable exception is Question R06-22-E, for which *no system succeeded* in retrieving the correct article. The relevant article for this question is Article 472, but many teams incorrectly selected Article 472-2, which outlines an exception to Article 472.

While Article 472-2 contains textually similar phrasing to the judicial decision in R06-22-E (e.g., “C may refuse performance of the obligation to A to the extent...” and “the new obligor may refuse to perform the obligation to the obligee to the extent that...”), the exceptional condition specified in Article 472-2 does not apply to the factual context of the question. Therefore the question cannot be entailed by Article 472-2 alone; Article 472 is the only valid basis for entailment. This case illustrates a limitation of models that compute similarity based only on textual overlap, without considering the argumentative structure, e.g., such as legal conditions and conclusions. Such models are more prone to failure in scenarios where surface-level similarity is misleading.

For the 18 questions with multiple relevant articles, retrieval performance has improved compared to previous years. As shown in Fig. 2, while precision remains relatively high, recall is comparatively lower for multi-article questions. This indicates that most systems are able to correctly identify one relevant article without including irrelevant ones, but often fail to retrieve the additional relevant article(s). This pattern is consistent with previous years. However, in 10 out of the 18 cases, recall exceeded 0.5, suggesting that at least one team succeeded in retrieving multiple relevant articles simultaneously. Despite these improvements, the overall difficulty of questions with multiple relevant articles remains significantly higher than that of single-article questions.

### Summary of Task 3

In this year, thanks to the improvement of LLMs, the best performance team and the second best performance teams achieved higher F2: i.e., higher than 0.8. Most of the questions that have single relevant articles are treated as easy questions for those systems.

It means there are now teams that achieve nearly perfect performance in the retrieval task based solely on the similarity to the problem statement.

Below, we summarize the remaining types of issues.

1. Cases where multiple articles are required as answers
  2. Handling of "mutatis mutandis"
    To use a provision that is applied "mutatis mutandis," it is necessary to identify both the article that specifies the application and the original article being referred to. However, such logic is not properly handled in most current methods (but it was included in earlier approaches [19]).
  3. The second required article is not the second most similar one
    When similarity alone is considered, articles are examined in order of their similarity scores. However, the second required article for the entailment is one that *complements what is missing* in the first article. Therefore, it cannot be determined without further consideration of entailment relations.
2. Handling of general provisions and exception clauses
  When the problem perfectly matches an exception condition, the article specifying the exception becomes the relevant provision. However, if the condition does not match exactly, the general provision becomes the basis for judgment. This also cannot be determined without further considering entailment.

Except for the relatively minor and formally manageable case of 1.a, these are all types of problems that cannot be properly discussed without considering the more challenging problem of entailment.

Given this situation, it seems that there is little point in continuing with retrieval tasks based solely on similarity to the problem statement, as in the current Task 3 setting. Details of the new task will be discussed later.

## Task 4 - Statute Law Textual Entailment and Question Answering

### Task Definition

Task 4 requires confirmation of entailment relationships between a given problem sentence and selected article sentences. Competitor systems should answer “yes” or “no” regarding the given problem sentences and given article sentences. Until COLIEE 2016, the competition used pure entailment tasks, where t1 (relevant article sentences) and t2 (problem sentence) were given. Participants can use any external data, except that they can not use the test dataset and/or something which could directly contain the correct answers of the test dataset, because this task is intended to be a pure textual entailment task. We also require the participants to make their system reproducible as per an open academic standard, i.e., they should describe which methods and what datasets were used to enable a reproducible result. To encourage a deeper analysis, we asked the participants to submit their outputs when using any fragment of the training dataset (H30-R05), in addition to the formal runs.

Task 4 evaluation was performed by accuracy:

4 $$\begin{aligned} accuracy= & \frac{\#\ of\ queries\ correctly\ confirmed\ as\ true\ or\ false}{\#\ of\ all\ queries} \end{aligned}$$

### Dataset

The training and test datasets are originally the same as for Task 3, described in Section 5.2.

### Approaches

We describe approaches for each team as follows, shown as a header format of **Team Name (number of submitted runs)**.

Runs with “*” as a suffix of the run names, which used 1) external services where its detailed architecture, training datasets, or model weights are not available, resulting in unreproducible outputs, or 2) using newer dataset which might include the gold standard answers. These are prohibited in our participation call from the academic perspective.

**Table 7:** Comparison of approaches for Task 4

Team	Encoder based LLM	Instruction based LLM	Ensemble	Model names
AIIRLab		+ few shot, CoT	+	Mistral-7B-Instruct-v0.2 Llama3-8B-Instruct
CAPTAIN		+ fine-tune	+	Qwen2-72B-Instruct) Flan-T5
IRNLPUI		+ CoT, fine-tune	+	Qwen-7B
JNLP		+ few-shot	+	Qwen2-72B-Instruct
KIS		+ few-shot	+	Llama3.1-Swallow-70B-v0.3 intfloat/multilingual-e5-large
KLAP		+		Llama3-70b
NOWJ		+	+	Qwen2-72B-Instruct
OVGU		+ fine-tune	+	Phi-3-medium-4k-instruct gemma−1.1-7b-it
RUG		+ one-shot	+	Llama3-8B-instruct
UA		+ pretrain, fine-tune	+	Llama3-70B

- *AIIRLab (2 runs)* [17] Using Mistral-7B-Instruct-v0.2^5^ in *AIIRMistral* and Meta-Llama-3-8B-Instruct^6^ in *AIIRLLaMA*, they consider three prompting techniques. The model is prompted to be an expert Japanese lawyer, who will decide if a given legal query can be entailed by given legal case. The model was prompted to answer yes or no, and the final result is a combination of the following three with majority voting: 1) zero-shot prompting, and just passed the query and candidate case 2) few-shot prompting, where a positive and negative entailment was passed to LLM, before passing the test query and 3) zero-shot chain-of-thought (CoT), where the model was asked to “think” or do its inference step-by-step.
- *CAPTAIN (3 runs)* [2] leveraged the knowledge and reasoning of the pre-trained LLM (Qwen2-72B-Instruct) to generate additional data, including extraction of atomic causal rules from law articles (cause $\rightarrow$ effect) to create new hypothesis pairs. They then combined the organizer’s data with their generated data (1 original:0.5 generated) and fine-tuned the Flan-T5 model using LoRA. *CAPTAIN1* employs a single fine-tuned Flan-T5 model with the best average test score on all test sets, **CAPTAIN2** and **CAPTAIN3** employ an ensemble (voter) mechanism by using the top three fine-tuned Flan-T5 models with different hyperparameters and inference prompts for prediction on all test sets.
- *IRNLPUI (3 runs)* [15] *UIRunCot (unofficial result due to their violation in their way using LLM) used Qwen with Chain of Thought processing (CoT), Graph of Thought (GoT), and Tree of Thought (ToT) prompting. Each method produced five-level labels; if “neutral” was returned, a secondary prompt forced a clearer decision. Final predictions were determined by majority vote. **UIRunFTune** fine-tuned the Qwen 7B model using three prompts, with predictions aggregated through ensemble voting. **UIRunLang** applied standard prompting with three prompts in both Japanese and English. If the translations matched in meaning (“Same Translation”), the English prediction was used. Otherwise, the Japanese version was retained.
- *JNLP (3 runs)* [10] Using Qwen/Qwen2-72B-Instruct, they gather responses along with explanations for each question using five distinct prompt patterns. Then **JNLP001** aggregates and analyzes the results from these five prompt patterns to determine the final outcome. **JNLP002** collects responses with explanations for each question using five distinct prompt patterns, leveraging all past training data. They then curate a corpus of correct answers to serve as few-shot examples. For each new query, they retrieve the three most relevant queries from the training corpus based on content similarity. These selected examples are used as the few-shot context, and the final result is then generated by the LLM. **JNLP003** fuse the answers of five prompts as an ensemble prompt to require LLM justify the final answer.
- *KIS (3 runs)* [11] They used Llama 3.1 Swallow 70B - v0.3 (tokyotech-llm/Llama−3.1-Swallow-70B-v0.3) which is additionally trained for the Jap-anese language. **KIS1** employs a zero-shot prompting approach. **KIS2** employs a few-shot prompting approach. They use an embedding model to select a few-shot sample set based on cosine similarity, ranking the most similar samples. **KIS3** employs a few-shot prompting approach similar to **KIS2**, but the selection process ensures that the number of samples per label is balanced while still prioritizing those with the highest cosine similarity. The submissions of **KIS2** and **KIS3** used multilingual-e5-large (intfloat/multilingual-e5-large) to select few-shot samples.
- *KLAP (2 runs)* used llama3:70b^7^ as the required LLM. *KLAP.H1* first extracts the ANGELIC structure from relevant articles and evaluates the query’s entailment for each base-level factor using zero-shot prompting with LLMs. Next, they generate Answer Set Programming (ASP) facts from this structure through LLMs. An ASP program is then executed to determine the label based on these facts. If a test instance produces an incorrect ASP format, they rerun the system to correct it. **KLAP.H2** use LLMs with zero-shot prompting to produce the predicted label.
- *LUONG (1 runs)* *LUONG (unofficial result due to their violation in their way using LLM) focuses on an in-depth study of the task and dataset to design optimal prompts. They employ a majority voting strategy to mitigate noise from the LLM and implement a continuous retry mechanism to handle format errors, ensuring the model generates the most accurate responses.
- *NOWJ (3 runs)* [9] Two of the *NOWJ* runs construct the few-shot prompting based on shared relevant articles and query’s semantic similarity. Note that legal knowledge is also added in the prompt. One of the runs used the Qwen-2-72B-Instruct model to generate answers; another one combined three models to generate answers, using majority voting. One of the three **NOWJ** runs use legal prompting with the “overthinking” keyword. Three models are used to generate answers and their answers were then combined using a majority voting approach.
- *OVGU (3 runs)* [16] created a “silver dataset” by prompting nine LLMs on the COLIEE 2025 training set using problem type definitions from Hoshino et al [3]. Models were asked to identify problem types, predict entailment labels, and provide reasoning. Responses were accepted if valid, correctly labeled, and included both problem type and reasoning. Using this data, **OVGU1** fine-tuned Phi-3-medium-4k-instruct, **OVGU2** fine-tuned gemma−1.1-7b-it, and **OVGU3** fine-tuned both. **OVGU3** applied majority voting across six models, including external LLMs. In case of a tie, Phi-3’s prediction was used. If unavailable, “N” was assigned.
- *RUG (3 runs)* [13] **RUG_V1** used Llama-3-8B-instruct^7^. **RUG_V2** additionally provided three guidelines that it should adhere to, based on input from a lawyer, and one-shot prompting selected from the training data based on Jaccard similarity. **RUG_V3** further used Black’s Law dictionary^8^ to add definitions to the prompt, created and used ADMs (knowledge representations) of legal article using o3-mini-2025-01-31.^9^
- *UA (3 runs)* [1] Their model is pretrained using the legal corpus provided in civil_code_en-1to724-2.txt. Fine-tuning is performed with the LoRA (Low-Rank Adaptation) method using a training dataset transformed into their instruction fine tuning format. During inference, for **UA1**, 15 sampling runs are executed per query with final Yes/No predictions determined by majority voting. Inference of **UA2** and **UA3** is performed with 15 sampling passes per query, and final predictions are derived by majority voting to address the inherent randomness of LLM outputs.
  Table 7 summarizes the approaches used by the submitted systems for Task 4, highlighting the key techniques employed across teams.

### Results and Discussion

Table 8 shows the COLIEE 2025 Task 4 formal run results. The Formal Run (R06) column shows the result of the COLIEE 2025 formal run using the latest Japanese legal bar exam (Year R06). The columns R02, R01, and H30 are the results using the past formal run datasets, which we required participants to submit in order to compare different datasets for reference. Note that these datasets were already made public as part of our training dataset.

The best runs by team **KIS** used LLMs with straightforward few-shot prompting. The second-best, by **CAPTAIN**, involved fine-tuned LLMs and ensemble methods, while **JNLP** used prompt-based ensemble techniques. A key distinction is that **KIS** used Japanese, whereas the others did not. Although Japanese versions were not superior in previous formal runs, recent LLM advancements may have shifted this. The combination of prompt language and base LLM likely contributed to improved results in this round.

These results suggest that future progress in Task 4 will depend on more robust prompt design, language-aware modeling choices, and improved handling of fine-grained legal reasoning.

**Table 8:** Evaluation results of submitted runs (Task 4). L: Dataset Language (J: Japanese, E: English). Runs with "**" as a suffix of the run names, which used 1) external services where its detailed architecture, training datasets, or model weights are not available, resulting in unreproducible outputs, or 2) using newer dataset which might include the gold standard answers. These are prohibited in our participation call from the academic perspective

Team	Submission ID	L	Formal Run (R06)		R02		R01		H30	
			Correct	Acc	Correct	Acc	Correct	Acc	Correct	Acc
# of Problems	–	–	73	1.0000	81	1.0000	111	1.0000	70	1.0000
No to All	BaseLine	–	36	0.5068	43	0.5309	52	0.5315	34	0.5143
KIS	KIS3	J	66	0.9041	71	0.8765	53	0.4775	59	0.8429
KIS	KIS1	J	64	0.8767	68	0.8395	51	0.4595	61	0.8714
KIS	KIS2	J	62	0.8493	69	0.8519	52	0.4685	62	0.8857
CAPTAIN	CAPTAIN2	E	60	0.8219	64	0.7901	N/A	N/A	N/A	N/A
IRNLPUI	UIRunLang	E/J	60	0.8219	N/A	N/A	N/A	N/A	N/A	N/A
JNLP	JNLP002	E	59	0.8082	N/A	N/A	N/A	N/A	N/A	N/A
JNLP	JNLP003	E	59	0.8082	N/A	N/A	N/A	N/A	N/A	N/A
CAPTAIN	CAPTAIN1	E	58	0.7945	64	0.7901	N/A	N/A	N/A	N/A
CAPTAIN	CAPTAIN3	E	58	0.7945	64	0.7901	N/A	N/A	N/A	N/A
UA	UA2	E	57	0.7808	N/A	N/A	N/A	N/A	N/A	N/A
UA	UA3	E	57	0.7808	N/A	N/A	N/A	N/A	N/A	N/A
JNLP	JNLP001	E	56	0.7671	N/A	N/A	N/A	N/A	N/A	N/A
KLAP	KLAP.H2	E	56	0.7671	N/A	N/A	N/A	N/A	N/A	N/A
UA	UA1	E	55	0.7534	69	0.8519	92	0.8288	58	0.8286
NOWJ	NOWJ.run1	E	54	0.7397	N/A	N/A	N/A	N/A	N/A	N/A
NOWJ	NOWJ.run2	E	54	0.7397	N/A	N/A	N/A	N/A	N/A	N/A
NOWJ	NOWJ.run3	E	54	0.7397	N/A	N/A	N/A	N/A	N/A	N/A
OVGU	OVGU1	E	54	0.7397	69	0.8519	81	0.7297	55	0.7857
RUG	RUG_V1	E	48	0.6575	51	0.6296	79	0.7117	41	0.5857
KLAP	KLAP.H1	E	48	0.6575	N/A	N/A	N/A	N/A	N/A	N/A
OVGU	OVGU3	E	46	0.6301	56	0.6914	71	0.6396	47	0.6714
RUG	RUG_V3	E	46	0.6301	62	0.7654	74	0.6667	45	0.6429
RUG	RUG_V2	E	45	0.6164	64	0.7901	64	0.5766	42	0.6000
AIIRLab	AIIRLLaMA	E	44	0.6027	53	0.6543	40	0.3604	36	0.5143
IRNLPUI	UIRunFTune	E	44	0.6027	N/A	N/A	N/A	N/A	N/A	N/A
OVGU	OVGU2	E	44	0.6027	76	0.9383	94	0.8468	62	0.8857
AIIRLab	AIIRMistral	E	41	0.5616	52	0.6420	42	0.3784	43	0.6143
LUONG	**LUONG01	E	63	0.8630	N/A	N/A	N/A	N/A	N/A	N/A
IRNLPUI	**UIRunCot	E	62	0.8493	69	0.8519	53	0.4775	60	0.8571

## New Task Proposal for Statute Law Component

As described in Sect. 4, since the retrieval task is based solely on similarity to the problem statement - such as the current Task 3 setting - there are almost no remaining challenges. So we plan not to include Task 3 in the next COLIEE competition.

On the other hand, the challenge of identifying the specific statutory provisions that are truly necessary for entailment, taking entailment into account, has not yet been sufficiently explored. Therefore, for the next year, we plan to introduce a new task that jointly performs statute retrieval and entailment, similar to the previous Task 5 of COLIEE 2021 [12]. Unlike the previous Task 5, participants will be required not only to submit the entailment results but also to explicitly specify the articles used for entailment. This evolution will help encourage the idea of explaining the results based on identified relationships between statutes and given statute cases.

For the evaluation of this new task, we intend to use accuracy, calculated only for systems that (i) include all the articles necessary for entailment (i.e., recall = 1) and (ii) produce correct entailment results. Given that the performance of Task 4 in COLIEE 2025 achieved an accuracy exceeding 0.9, it can be inferred that systems with a stronger ability to retrieve the necessary articles for entailment are evaluated as better-performing systems under this task framework. However, since this approach may overly reward systems that simply include an excessive number of candidate articles in their retrieval results, we will consider incorporating evaluation measures such as precision and F2-score used in the previous Task 3 settings.

## Pilot tasks - Legal Judgment Prediction and Rationale Extraction in Japanese Tort Cases

### Task Definition

**Fig. 3:**
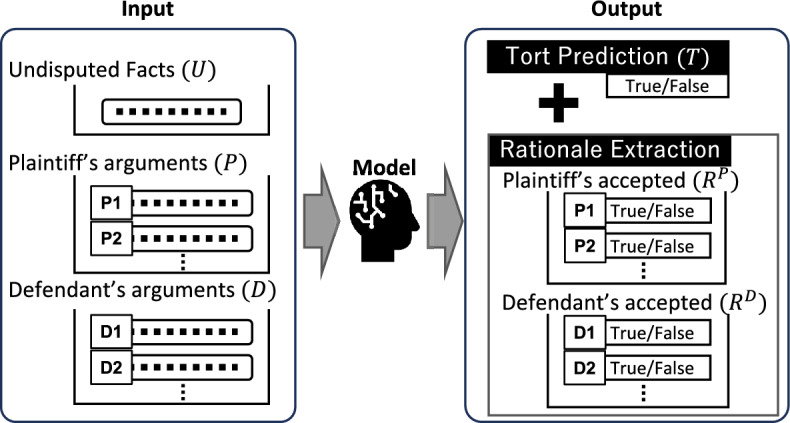
Pilot tasks overview, revised Fig. 1 from [18]

From this iteration of COLIEE, we introduced new pilot tasks, involving cases about torts of Japan (Civil Code, Art. 709).^10^ The code affirms a tort as a negligent or intentional infringement of rights or legal interests that cause a plaintiff to suffer loss or harm. Torts play an important role in disputes on the Internet, for example, cases of defamation and privacy infringement on social media. A tort case involves two parties: plaintiffs and defendants. Plaintiffs are claimants of the case, arguing that a defendant’s action is a tort, while defendants contest the plaintiffs’ arguments.

The new tasks serve as a sandbox for researchers to develop legal judgment prediction models using real court case data from Japanese courts. Figure 3 shows the overview of the tasks. Our pilot tasks consist of two tasks: Tort Prediction (TP) and Rationale Extraction (RE). TP is a task to predict whether a tort is affirmed or not (*T*, a Boolean value), given undisputed facts (*U*) and arguments from the parties, where *P* is a list of arguments from plaintiffs and *D* is a similar list from defendants. Undisputed facts are not disputed by either party or agreed upon by both parties, so that they are neutral and appear only in input. *T*, the final output on a tort, is based on the arguments that are accepted by the judge. So in summary, the accepted arguments can be considered rationales for the final output (*T*). The RE task identifies the accepted arguments for the parties, $R^{P}$ for plaintiffs and $R^{D}$ for defendants. They are lists of Boolean values, denoting accepted arguments as True in the parties’ arguments (*P* and *D*). Our tasks take (*U*, *P*, *D*) as input and output (*T*, $R^{P}$, $R^{D}$).

During the competition, we provided an automated leaderboard system, which evaluated each submission and returned metrics on the test split. The leaderboard system aimed to provide fast instant evaluation, allowing participants to develop and improve their models more dynamically.

Dataset statistics for the pilot tasks are described in Sect. 7.2. Official evaluation metrics used for the leaderboard are described in Sect. 7.4.

### Dataset

We constructed the dataset for the pilot tasks from the Japanese Tort-case Dataset (JTD) [18]. The dataset comprises tort-related judgment documents from the first instances of Civil Code cases in lower courts in Japan. All the documents are written in Japanese. The training split of the dataset consists of 6508 TP instances with 47,689 arguments for RE, and the test split consists of 812 TP instances with 5945 arguments for RE.

### Approaches

In this pilot task, participants could submit their runs up to 20 times to our automated leaderboard system. For valid submissions to formally participate in the competition, participants had to select up to three of their leaderboard runs, provide system descriptions for each, and submit their summary paper. During the competition period, the leaderboard received 14 runs for TP and 15 runs for RE. Of these, 10 runs^11^ from four teams were registered as valid submissions for each task.

In the following, we provide a summary of the approaches proposed by the teams that submitted their system description papers.

- *CAPTAIN (1 run for TP, 1 run for RE)* [2] took a straightforward approach of fine-tuning a generative LLM to solve both TP and RE. They used Linkbricks-Horizon-AI-Japanese-Pro-V5-70B for their base model, which was the best-performing model according to the Open Japanese LLM Leaderboard [7].
- *KIS (3 runs for TP, 3 runs for RE)* [5] submitted three runs (KIS4, KIS5, KIS6). They fine-tuned modernbert-ja-130 m, which is a variant of ModernBERT, an encoder-based model. It could accept a longer input sequence than the original BERT. **KIS4** was their best-performing single fine-tuned model. They also implemented ensemble-based approaches. In **KIS5**, they fine-tuned models with different sets of hyperparameters and created an ensemble of the top five models according to their development set, which was a part of the official training set. In **KIS6**, they also fine-tuned five models using the same set of hyperparameters in a five-fold cross-validation manner and the ensembles of those models.
- *NOWJ (3 runs for TP, 3 runs for RE)* [9] used a hierarchical transformer architecture with modernbert-ja-310 m for word-level encoding and a Transformer for span-level encoding. TP was solved as binary classification, and RE as sequence labeling with a Conditional Random Field layer to capture claim relationships. **System1** used this base setup. **System2** added heuristic post-processing to adjust the number of accepted claims. **System3** applied a clustering-based method to group claims semantically, using DeepSeek-V3 to assess groups for final TP and RE predictions.
- *OVGU (3 runs for TP, 3 runs for RE)* [16] used a two-step pipeline, solving RE with LLMs and then TP using a rule-based scoring function based on training-set correlation analysis. For RE, their prompt included a task description, the argument, and a summary of undisputed facts. **OVGU1** used aya-expanse-8b to directly predict RE labels. **OVGU2** and **OVGU3** first generated the summaries with aya-expanse-8b, then used phi-4 and gemma3-12b, respectively, for claim classification. TP in all runs was handled by the same scoring function.

### Results and Discussion

**Table 9:** Pilot task tort prediction results

Run	Team	Accuracy
JAIST-LJPJT25	CAPTAIN	**0**.**765**
KIS5	KIS	0.713
KIS6	KIS	0.713
KIS4	KIS	0.697
system2	NOWJ	0.671
system1	NOWJ	0.638
system3	NOWJ	0.597
OVGU2	OVGU	0.553
OVGU3	OVGU	0.532
OVGU1	OVGU	0.515

**Table 10:** Pilot task Rationale Extraction results. Bold values indicate the highest performance

Run	Team	F1 (All)	F1 (*P*)	F1 (*D*)
KIS5	KIS	**0**.**712**	0.740	**0**.**673**
JAIST-LJPJT25	CAPTAIN	0.706	**0**.**743**	0.663
system2	NOWJ	0.692	0.733	0.640
KIS4	KIS	0.682	0.725	0.631
system1	NOWJ	0.681	0.724	0.626
KIS6	KIS	0.673	0.718	0.605
OVGU1	OVGU	0.657	0.696	0.610
system3	NOWJ	0.559	0.446	0.637
OVGU2	OVGU	0.486	0.516	0.450
OVGU3	OVGU	0.316	0.338	0.290

For TP, we use accuracy as our official metric, based on whether the True/False label for the decision on tort (*T*) was correctly predicted. For RE, our official metrics is F1, concerning the True label for *P* and *D*.

5 $$\begin{aligned} Prec.= & \frac{num\ of\ arguments\ correctly\ predicted\ as\ True}{num\ of\ arguments\ predicted\ as\ True} \end{aligned}$$

6 $$\begin{aligned} Recall= & \frac{num\ of\ arguments\ correctly\ predicted\ as\ True}{num\ of\ arguments\ whose\ gold\ labels\ are\ True} \end{aligned}$$

7 $$\begin{aligned} F1= & \frac{2 \times Precision \times Recall}{Precision + Recall} \end{aligned}$$

We first calculate the F1 for each tort instance, then average it over all tort instances. We also report F1 values for performance by parties (plaintiffs *P* and defendants *D*) as auxiliary metrics. Tables 9 and 10 show the results of TP and RE on the test set. They are sorted in descending order of accuracy and F1 (All), respectively. The best run for TP was JAIST-LJPJT25 by CAPTAIN. For RE, KIS5 by KIS was the best run.

Runs from KIS and system1 and system2 from NOWJ utilized masked language models (modernBERT), which follow a transformer-encoder architecture and were therefore trained and applied as classifiers. In contrast, runs from CAPTAIN and OVGU used autoregressive generative language models and solved the tasks in a text-to-text inference setting.

JAIST-LJPJT25 showed a significantly better performance than the 2nd-ranked run by KIS5 in TP. The model used by JAIST-LJPJT25 was one of the best-performing language models in the general Japanese benchmark. This result confirms the importance of domain-independent performance in solving Japanese legal domain tasks. In RE, there are considerable gaps in the sizes of the language models used between the two closely competing runs: JAIST-LJPJT25 and KIS5. The KIS5’s fine-tuning 130 M model approach won against the JAIST-LJPJT25 with the 70B model. This result shows that an encoder-based language model is still competitive against the larger decoder-based generative language models with more parameters.

In the 2025 edition of the task, the established state-of-the-art scores are already strong, yet there is still room to improve. The top-performing approaches were fine-tuning LLMs pre-trained on general domains and did not utilize domain-specific features. It is natural to assume that future approaches will explore enhancing legal judgment prediction by integrating more legal domain expertise.

## Conclusion

We presented an overview of the systems and results submitted to the COLIEE 2025 competition, covering multiple tasks in legal information retrieval, entailment, and judgment prediction. Across tasks, the results highlight the continued importance of combining robust retrieval strategies with effective language modeling, as well as the growing role of large language models in addressing complex legal reasoning challenges.

The data used in the COLIEE competition are publicly available at https://coliee.org.

Looking forward, we plan to further improve dataset quality and task design so that future editions of COLIEE more accurately reflect real-world legal problems. In particular, we aim to place greater emphasis on explicit error analysis and explainability, enabling a deeper understanding of system failures and supporting the development of more transparent and debuggable legal AI systems.

## Acknowledgements

This research was supported by the Canadian Natural Sciences and Engineering Research Council (NSERC) [including funding reference numbers RGPIN-2022-03469 and DGECR-2022-00369], Alberta Innovates and JST PRESTO Grant Number JPMJPR236B.

## Appendix A: Dataset Deduplication Process

### Problem Identification

The original Task 1 dataset contained 2,159 test cases and 7,350 training cases. During preparation for publication, we identified duplicate documents-cases with identical content stored as separate files with different IDs and potentially different label sets. These duplicates created two significant issues:

- During evaluation, systems could retrieve correct document content but receive false negatives if they retrieved the "wrong" duplicate ID
- During training, machine learning systems encountered identical content with conflicting labels, hindering proper learning

### Deduplication Solution

We performed comprehensive duplicate detection and consolidation:

- Identified all cases with identical content across the dataset
- Selected canonical versions using alphabetically first IDs
- Merged all associated labels from duplicate instances
- Removed duplicate files and updated all JSON references to canonical versions

### Impact Analysis

Evaluation of existing submissions against the cleaned dataset showed:

- Test Dataset: 2159 $\rightarrow$ 1870 files (289 duplicates removed)
- Train Dataset: 7350 $\rightarrow$ 6835 files (515 duplicates removed)
- Team rankings remained stable with no changes to competitive balance
- Most teams saw modest 5–10% F1 improvements, distributed proportionally
- No significant changes to relative performance gaps between teams

The cleaning process maintained ranking stability while ensuring that retrieving correct document content is properly credited regardless of which duplicate ID was originally present. All results in this paper reflect evaluation on the cleaned dataset.

## Footnotes

1 We originally distributed 83 questions, but excluded 9 questions that required revised Civil Code articles not included in the training set and excluded 1 question whose English translation is not correct.

2 The submission from NOWJ arrived just after the official deadline. Therefore, it is treated as an unofficial submission and was excluded from the competition.

3 JNLP’s long list corresponds to the output of their first-stage retrieval. In some cases, the final selected articles are not included in the top-ranked results. Therefore, we exclude MAP for JNLP due to the differing nature of their output.

4 Due to space limitations, we select 26 questions for which whose average F2 score was less than 0.9.

5 https://huggingface.co/mistralai/Mistral-7B-Instruct-v0.2.

6 https://huggingface.co/meta-llama/Meta-Llama-3-8B-Instruct

7 https://ollama.com/library/llama3:70b.

8 https://github.com/LexPredict/lexpredict-legal-dictionary/ (second edition)

9 https://platform.openai.com/docs/models/o3-mini.

10 https://www.japaneselawtranslation.go.jp/en/laws/view/3494#je_pt3ch5at1

11 We did not receive a paper from team Omega, thus we exclude their result in the following discussion. All runs, including informal submissions, are available at https://ljpjt25.coliee.org/
